# Introduction to the Special Issue: New Advances in the Research of Antioxidant Food Peptides

**DOI:** 10.3390/foods9121810

**Published:** 2020-12-07

**Authors:** Lourdes Amigo, Blanca Hernández-Ledesma

**Affiliations:** Institute of Food Science Research (CIAL, CSIC-UAM, CEI UAM + CSIC), Nicolás Cabrera 9, 28049 Madrid, Spain; lourdes.amigo@csic.es

During cell metabolism, oxygen is partially reduced to reactive oxygen species (ROS) that play a physiological role in cellular processes, including proliferation, cell cycle and death, and signal transduction. However, ROS are responsible for attacking cell nucleophilic centers, causing lipid peroxidation, protein oxidation, and genetic alterations, including DNA damage, mutations, epigenetic changes, and genomic instability. Fortunately, the human body is equipped with an effective defense system to neutralize the toxicity of ROS. However, an imbalance provoked by either an over-production of ROS or a defect in the cellular defense system results in a state known as oxidative stress. This status and its subsequent damages to vital cellular components have been associated with numerous severe chronic disorders, such as cardiovascular and neurodegenerative diseases, diabetes, metabolic syndrome, intestinal inflammatory diseases, and cancer. In addition, oxidation reactions are responsible for food deterioration during processing and storage. In spite of their remarkable effectiveness, the endogenous antioxidant systems are not sufficient and humans are dependent on dietary antioxidants to maintain ROS concentrations at low levels. A number of natural antioxidants have been revealed as potential preventative/therapeutic agents against oxidative stress. Among them, peptides from animal and vegetal food sources have attracted attention because of the plentiful evidence of their in vitro antioxidant properties. In addition to their potential as safer alternatives to synthetic antioxidants used to prevent oxidative reactions in foods, antioxidant peptides can also act by reducing the risk of numerous oxidative stress-associated diseases. Furthermore, peptides can act synergistically with non-peptide antioxidants, enhancing their protective effect.

This Special Issue of the *Foods* journal includes seven outstanding papers describing examples of the most recent advances in the antioxidant peptide research. It begins with a group of papers describing the antioxidant potential of vegetal food-derived hydrolyzates and peptides. The review of Olivares-Galván et al. [1] summarized the existing evidence on antioxidant peptides released from fruit residues, focusing on the current techniques used in their extraction, purification, fractionation, and identification, the strategies followed to allow the peptides’ release from source protein, as well as the assays used to determine their antioxidant activity. Two research articles described the potential of two vegetal proteins as sources of antioxidant peptides. Thus, the study of Kusumah et al. [2] aimed at identifying the mung bean (Vigna radiata) albumin sequences with antioxidant capacity by both in silico and in vitro assays. The hydrolysates produced by thermolysin showed high antioxidant potential in terms of ferrous ion chelating and ORAC values because of the high hydrophobicity and low molecular mass of the released peptides. In the study of Esfandi et al. [3], hydrolyzates from oat bran protein with Alcalase^®^, Flavourzyme^®^, papain or Protamex^®^ showed the ability to protect hepatic HepG2 cells against oxidative damage by reducing ROS levels and caspase-3 activity, and increased glutathione concentration and antioxidant enzymes activity.

The following articles focused on animal protein sources of antioxidant peptides. First, the review of Gilmartin et al. [4] described the potential role exerted by whey protein, hydrolyzates or peptides in the modulation of sarcopenic biomarkers in myoblast cell lines, and in aged rodents and humans. The human intervention trials have shown that a daily dietary supplementation of 35 g of whey is likely to improve sarcopenic biomarkers, improving muscle mTOR signaling in the elderly, although exercise appears to have the greatest benefit for old muscle. Kleekayai et al. [5] hydrolyzed bovine whey protein concentrate with Debitrase^TM^ and FlavorPro^TM^ under pH-stat and non pH-controlled conditions, evaluating the impact of hydrolysis conditions on the physicochemical and antioxidant activities of the released hydrolyzates. These authors demonstrated that hydrolyzates generated under pH-stat conditions displayed higher radical scavenging activities than those shown by non-pH-controlled conditions. Moreover, a more pronounced impact of conditions in the biochemical assays compared to the cellular antioxidant assay was observed. In the study of Amigo et al. [6], an integrated approach combining in silico and in vitro assays was used, confirming the multifunctionality of milk and meat protein-derived peptides that were similar to or shared amino acids with previously described opioid peptides. Fifteen of twenty-seven assayed peptides were found to exert two or more activities, with angiotensin-converting enzyme (ACE) inhibitory, antioxidant, and opioid being the most commonly found. Four fragments, RYLGYLE, YLGYLE, YFYPEL, and YPWT, demonstrated ACE-inhibitory and antioxidant activities, and also protected Caco-2 and macrophage RAW264.7 cells against chemical-induced oxidative damage. Finally, the review of Benedé and Molina [7] summarized the current knowledge on the antioxidant activity of chicken egg proteins and their derived peptides. The main process for obtaining these bioactive peptides from their source proteins is enzymatic hydrolysis using enzymes and/or processing technologies, such as heating, sonication or a high-intensity-pulsed electric field. Different in vitro assays have been used to evaluate the mechanisms of action of egg bioactive peptides, the in vivo effects of which have been confirmed by both cell culture assays and animal models.
